# Extrinsic motives to encourage extracurricular research activities: a reminder call to medical schools in Saudi Arabia

**DOI:** 10.5116/ijme.58bb.e1d6

**Published:** 2017-03-23

**Authors:** Ahmed Abu-Zaid, Leenh O. BaHammam, Talal M. Hijji, Ismail M. Shakir, Abdulaziz M. Eshaq, Mohammed Alawadi, Abdulrahman A. Al-Khateeb, Tehreem A. Khan, Akef Obeidat, Khaled Alkattan

**Affiliations:** 1 College of Medicine, Alfaisal University, Riyadh, Saudi Arabia

**Keywords:** Extrinsic motives, extracurricular, research activities, medical schools, Saudi Arabia

## Introduction

At present, practice of clinical medicine is predominately dependent on proper application of "evidence-based medicine", which is in turn largely dependent on proficient acquisition of research skills.^1^ In fact, in the 21^st^ century, research proficiency continues to be regarded as an indispensable skill for all forthcoming medical graduates.^2^ Moreover, graduating clinically-qualified and research-trained medical professionals emerges as a crucial healthcare necessity. In line with the above-mentioned notions, there is a rapidly mounting propensity towards incorporating structured scientific research training into undergraduate medical education.^1^^,^^3^

There are two primary modes of integrating structured scientific research training into undergraduate medical education: (A) curricular, and (B) extracurricular. In developing and under-developing countries (including Saudi Arabia), optimal implementation of curricular research training remains fairly unachievable by medical schools.^4^^,^^5^ This problem is further compounded by the fact that extracurricular research activities remain largely unpopular by medical students. The latter phenomenon may be attributed, at least partly, to lack of adequate extrinsic motives at such medical schools.

For medical students to productively participate in extracurricular research activities, intrinsic and extrinsic motives should be present. Intrinsic motives include: developing one’s research-specific and transferable skills, fulfilling an inquisitiveness to investigate scientific matters of interest, strengthening one’s resume and mapping out a strategy to ease acceptance into postgraduate education programs.^2^ Although intrinsic motives are important, they should be reinforced by complementary extrinsic motives in order to maximize engagement and optimize productivity in extracurricular research activities.

Medical schools bear the foremost responsibility of providing extrinsic motives which push students towards excelling in extracurricular research activities. Unfortunately, despite playing pivotal roles, extrinsic motives are often neglected by medical schools in Saudi Arabia and are therefore, not paid their due attention.

Herein, we briefly present our perspectives on potential extrinsic motives which ought to be in place at medical schools in Saudi Arabia, with the ultimate aim of encouraging medical students to take part in extracurricular research activities. We broadly divide these extrinsic motives into: (A) financial and (B) non-financial rewards.

### Financial rewards

There is no doubt regarding financial rewards being one of the most enticing incentives for medical students to actively take part in research. This is particularly true for those who culminate their research endeavors in peer-reviewed publications. Not only do these student-authored publications add to the medical school’s pool of scholarly publications,^6^ but they also open doors for publishing medical students to achieve two main goals. The first goal is to receive individual financial research excellence awards on both national and international levels, which helps to reward one’s hard work and provide a compelling sense of accomplishment. The second goal is to increase the likelihood of bringing external research grants to the medical school, and thus promoting it as a growing research-intensive institution. Overall, the financial rewards can be regarded as a form of reciprocal appreciative exchange for students’ diligence. Moreover, they are expected to positively contribute to establishing a continuum of extracurricular research activities and mutual student-institution benefits in the short- and long-term.

Financial rewards can take several forms. Such forms include "cash awards" which are more likely to prompt medical students to further pursue and maintain the regularity of their participation in extracurricular research activities. Another form is "scholarship financial support" — that is, discounts on academic tuition fees. This reward is anticipated to impact favorably on students’ research productivity, offer a sense of emotional satisfaction and positively encourage students to excel in their extracurricular research activities. Furthermore, another kind of a satisfactory financial reward comes in the form of a "research travel grant". The purpose of this reward is to disseminate research findings in regional and international scientific meetings which carry the additional perks of being around, and learning from, eminent researchers from around the world.

Whilst the concept of financial rewards is positive and meant to encourage extracurricular research activities, it should not, intentionally or unintentionally, cultivate a culture where unethical research antics run rampant amongst medical student researchers, as money should not be thought of as the ultimate end goal. Such unethical research practices may include fraud, fabrication, plagiarism, low-quality work, and most commonly, authorship disintegrity in all its forms in order to produce publications and receive financial awards.^7^ Therefore, medical schools should take into consideration this potential matter and propose effective mechanisms to maintain a controlled environment where research means are guided by ethical guidelines. This can be achieved through establishment of an ethical research committee dedicated towards monitoring extracurricular research activities, as well as through structured curricular and extracurricular awareness lectures so that students develop proper ethical research attitudes.

### Non-financial rewards

Academic recognition of the students’ research activities is one of the most important non-financial rewards, as it provides a great sense of satisfaction and instills a positive driving sense of determination to excel further in the future. Forms of academic recognition include acknowledgement of students’ research achievements during medical schools’ freshman convocations, graduation ceremonies, on-campus research symposia and on social media.^8^ Additional forms of academic recognition comprise inviting active student researchers to take part in administrative-level college meetings whereby they may be included, albeit to a regulated extent, in decision-making regarding on-campus research activities. Akin to an "Academic Honors’ List" for high academic achievers, the creation of a "Research Honors’ List" may be a plausible method of appreciation of active student researchers. Lastly, a somewhat debatable non-financial reward is considering students for extra bonus marks (whether in elective or core courses) as a reward for their valuable student-authored publications.

Although academic recognition is a remarkable way to boost student motivation towards extracurricular research pursuits, care needs to be taken to ensure that such recognition does not, intentionally or unintentionally, help support a practice of arrogance, perkiness, immoral pride or nurture a "showing off" culture amongst the student body. Academic recognition for research endeavors should be perceived as a reward for diligence, commitment to achieve goals and research excellence. It must not be seen as the sole aim and outcome of such pursuits. Such negativity can be addressed through the implementation of well-structured curricular and extracurricular series of lectures/workshops aimed at developing positive attitudes towards scientific research and publication.

## Conclusions

Although engagement of medical students in extracurricular research activities should be chiefly propelled by intrinsic motives, extrinsic motives — mainly provided by medical schools — should be looked upon as equally important. These extrinsic motives can be broadly categorized into financial and non-financial rewards. Medical schools should stimulate their medical students to undertake actives roles in extracurricular research activities and contribute scholarly publications.

### Conflicts of Interest

The authors declare that they have no conflict of interest.
